# A Comparative Study of Primary Adenoid Cystic and Mucoepidermoid Carcinoma of Lung

**DOI:** 10.3389/fonc.2018.00153

**Published:** 2018-05-15

**Authors:** Vivek Kumar, Parita Soni, Mohit Garg, Abhishek Goyal, Trishala Meghal, Stephan Kamholz, Abhinav Binod Chandra

**Affiliations:** ^1^Department of General Internal Medicine, Brigham and Women’s Hospital, Boston, MA, United States; ^2^Department of Internal Medicine, Maimonides Cancer Center, New York, NY, United States; ^3^Department of Hematology and Oncology, Maimonides Cancer Center, New York, NY, United States; ^4^Department of Hematology and Oncology, Yuma Regional Medical Center Cancer Center, Yuma, AZ, United States

**Keywords:** pulmonary adenoid cystic carcinoma, pulmonary mucoepidermoid carcinoma, salivary gland tumor, primary salivary gland-type tumor, Surveillance Epidemiology and End Results, epidemiology, survival, incidence rate

## Abstract

**Background:**

Pulmonary mucoepidermoid carcinoma (PMEC) and pulmonary adenoid cystic carcinoma (PACC) are the two major types of primary salivary gland-type (PSGT) lung cancers. The demographic profile, clinicopathological features, and predictors of survival as an overall group have not been described for PSGT cancers of lung.

**Methods:**

In this study, we analyzed demographic, clinical, and survival data from 1,032 patients (546 PMEC and 486 PACC) who were diagnosed of PSGT lung cancer in the Surveillance, Epidemiology and End Results database from 1973 to 2014.

**Results:**

The PSGT constituted 0.09% of all lung cancers with age-adjusted incidence rate of 0.07 per 100,000 person-years and change of −32% from 1973 to 2014. The incidence of PMEC was slightly higher than PACC but there were no differences in the age and sex distribution. PACCs (55%) were significantly higher at trachea and main bronchus while PMECs were more common at peripheral lungs (85%). Most of the tumors were diagnosed at an early stage and were low grade irrespective of histology. As compared to PMEC, significantly higher number of patients with PACC underwent radical surgery and received adjuvant radiation. The 1- and 5-year cause-specific survival was 76.6 and 62.8%, respectively. On multivariate analysis, the survival was affected by age at diagnosis, tumor stage, histological grade, period of diagnosis, and surgical resection. The histology showed strong interaction with time and hazard ratio of patients with PACC was significantly worse than patients with PMEC only after 5 years.

**Conclusion:**

The incidence of pulmonary PSGT cancer is 7 cases per 10 million population in the United States and is decreasing. There was no difference between demographic profile of patients with PMEC and PACC but pathological features were diverse. The difference in the survival of patients with the two histological types surfaced only after 5 years when survival of patients with PMEC was better than PACC.

## Background

Primary salivary gland-type tumors of the lung (PSGT) is a distinct category of lung tumors according to the World Health Organization (WHO) classification of lung cancer (1). These malignancies constitute <1% of lung cancer (2, 3). The two major subtypes of PSGT are pulmonary adenoid cystic carcinoma (PACC) and pulmonary mucoepidermoid carcinoma (PMEC). However, WHO classification of PSGT is non-specific and can include any tumor found in the salivary glands, cases of epithelial myoepithelial carcinoma, clear cell carcinoma, acinic tumors, mixed carcinoma, and carcinoma ex pleomorphic adenoma have been reported so far (4–9).

Data on PACC and PMEC are derived from case series and retrospective studies (10–13). The true incidence of PSGTs is unknown in part due to their rarity. There are differences in the demographic and tumor characteristics of PACC and PMEC; however, reports are frequently conflicting. Some studies have suggested that patients with PACC have a poor prognosis when compared to those with PMEC because PACC often presents in an advanced stage at the time of diagnosis and exhibits infiltrative behavior (11, 14). Analyses of PACC and PMEC based on the National Cancer Institute’s Surveillance, Epidemiology and End Results (SEER) database did not identify differences in the demographic and tumor characteristics of pulmonary PACC and PMEC, nor was the impact of histopathology on survival assessed (15, 16). The present study was designed to compare the epidemiology and tumor characteristics of pulmonary PACC and PMEC and to analyze the survival impact of histology, independent of other demographic and tumor-related factors of patients in the SEER database.

## Methods

### Data Sources and Selection of Patients

The Maimonides Medical Center Institutional Review Board (IRB) has deemed that studies which utilize deidentified, publicly available data (current study) to be exempt from IRB review.

We included patients aged above 15 years old from the SEER database during the period 1973–2014 who had PACC and PMEC localized to the trachea, main bronchus, and lungs (codes 8200 and 8430, respectively, in version 3 of the International Classification of Diseases for Oncology; ICD-O-3 with Site codes C 30.9–31.8) from 1973 to 2014. Patients were excluded if they were diagnosed at the time of autopsy or did not have histologic confirmation of diagnosis.

The SEER database details patient characteristics including sex, age at diagnosis, ethnicity, state and county of residence, methods of diagnostic confirmation, stage at the time of diagnosis, primary tumor site, month and year of diagnosis, first course of treatment (radiation/surgery), use of chemotherapy, survival status (alive or dead), and the month and year of the last known follow up, which is linked to National Death Index data from the National Center for Health Statistics. The cause of death was recorded as described in ICD-10.

We assessed associations among the demographic, clinical, and pathologic characteristics of patients and their survival time. Demographic variables included gender, age at diagnosis, year of diagnosis, race/ethnicity, and the geographic area as identified by its Contract Health Service Delivery Area designation. The year of diagnosis was utilized as a categorical variable with the following time intervals: 1973–1999 and 2000–2014. Race/ethnicity was classified as white and non-white.

Data were available on anatomic location of the tumor in the lung, radiation therapy, surgery, sequence of radiation and surgery, and survival time. Data on radiation therapy included external beam radiation, radioisotopes, and other modalities. Radiation therapy was analyzed as a binomial variable, based on whether patients had received any form of radiation therapy Surgical therapy was defined by the site-specific lung surgery codes.

## Statistical Analysis

Categorical variables were presented as counts and percentages and were compared utilizing chi-square tests. Continuous variables were presented as median and interquartile range and were compared using the Mann–Whitney *U* test. Survival analysis was conducted using Kaplan–Meier method and the log rank test was used to compare the survival curves. For multivariate analysis, extended Cox’s proportional hazards (CPH) regression was applied, after checking for fulfilment of the proportional hazard assumption. Histology data did not fulfill the proportional hazard assumption and thus was applied as time-dependent variable. Due to missing information for several variables, variables were adjusted in a stepwise manner in the following two models:

*Model I*: age + sex + ethnicity + anatomical site + year of diagnosis + surgery, and/or radiotherapy + interaction histology: time.
*Model II*: variables in model I + stage + histological grade + tumor size.

The strength of association between each predictor variable and survival was expressed as a hazard ratio (HR), and was presented along with a 95% confidence interval (95% CI). All tests were two sided, and a “*p*” value <0.05 was considered statistically significant. All analyses were performed using Statistical Package for the Social Sciences (SPSS) software (version 23; SPSS Inc., Chicago, IL, USA), SEER*Stat software (version 8.3.4; surveillance research program, National Cancer Institute, Bethesda, MD, USA) and “R” version 3.4.1.

## Results

### Baseline Characteristics of Study Population

Table 1 delineates the characteristics of 1,032 patients included in the SEER database (from 1973 to 2014) who had a diagnosis of one of the two major subtypes of PSGT, PMEC, and PACC stratified for histopathologic subtypes. The majority of the patients were white, in the sixth decade of life [median age 57.5 years (IQR 42–67.5)] with a slight female predominance (51 vs. 49%). During this period, a total of 1,154,608 primary lung tumors were included in the SEER database; thus, PACC and PMEC represented 0.087% of all lung tumors.

**Table 1:** Characteristics of the study population with diagnosis of primary pulmonary adenoid cystic carcinoma (ACC) and mucoepidermoid carcinoma (MEC).

Variables	Total population*n* = 1,032	ACC *n* = 486	MEC *n* = 546	*P* value
**Demographics**				
Median age in years (IQR^a^)	57.5 (42–67.5)	57.5 (47.5–67.5)	57.5 (37.5–67.5)	0.96
**Ethnicity**				
White	820 (79)	392 (81)	428 (78)	0.37
Non-White	212 (21)	94 (91)	118 (22)	
**Gender**				
Male	505 (49)	249 (51)	256 (47)	0.16
Female	527 (51)	237 (49)	290 (53)	
**Geographic region**				
East	339 (33)	145 (30)	194 (36)	
Northern plains	146 (14)	83 (17)	63 (11)	**0.03**
Pacific Coast	480 (47)	222 (46)	258 (47)	
South-west	67 (6)	36 (7)	31 (6)	
**Tumor sequence**				
1 primary or first of 2 or more	866 (84)	393 (72)	473 (87)	**0.012**
>2	166 (16)	93 (28)	73 (13)	
Median size in mm (IQR)	29 (18–40)	30 (20–50)	25 (17–38)	**0.007**

*^a^Interquartile range*. *Values in bold significant at p < 0.05*.

### Incidence Rate and Trend

**Figure 1:**
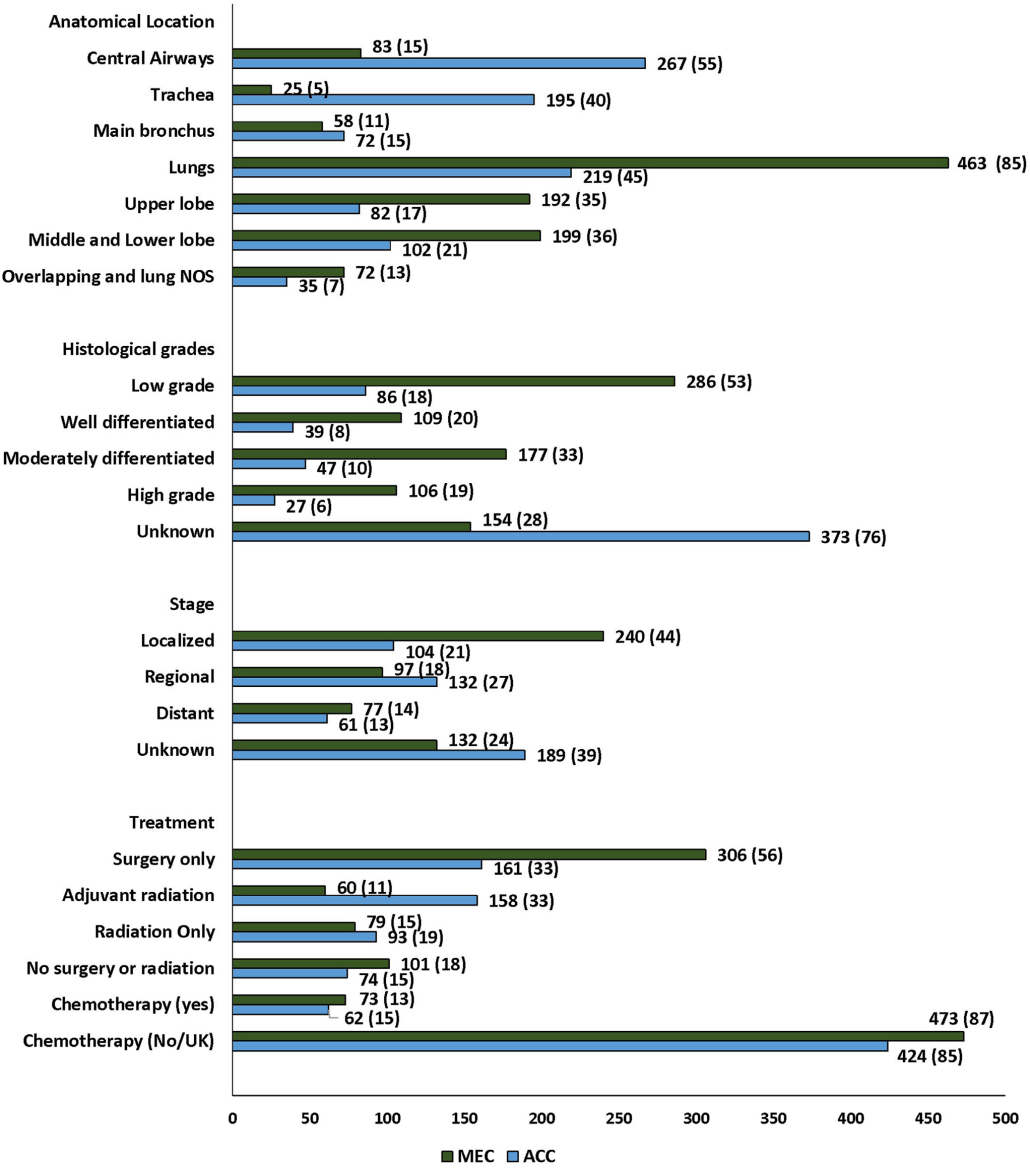
Characteristics and distribution of primary adenoid cystic carcinoma and mucoepidermoid carcinoma of lung in patients diagnosed in the Surveillance Epidemiology and End Results database.

The overall age-adjusted (to the 2000 US standard population) incidence rate (AAIR) of PSGT in the SEER database during 1973–2014 was 0.073 per 100,000 person-years. The AAIR of PACC and PMEC was 0.034 and 0.038 per 100,000 person-years, respectively. The study of trend revealed decrease in the overall AAIR in the study period from 1973 to 2014 with percent change of −32.24 and annual change of −1.5% (95% CI −2.3 to −0.83) (Figure 1).

### Patient Characteristics

**Figure 2:**
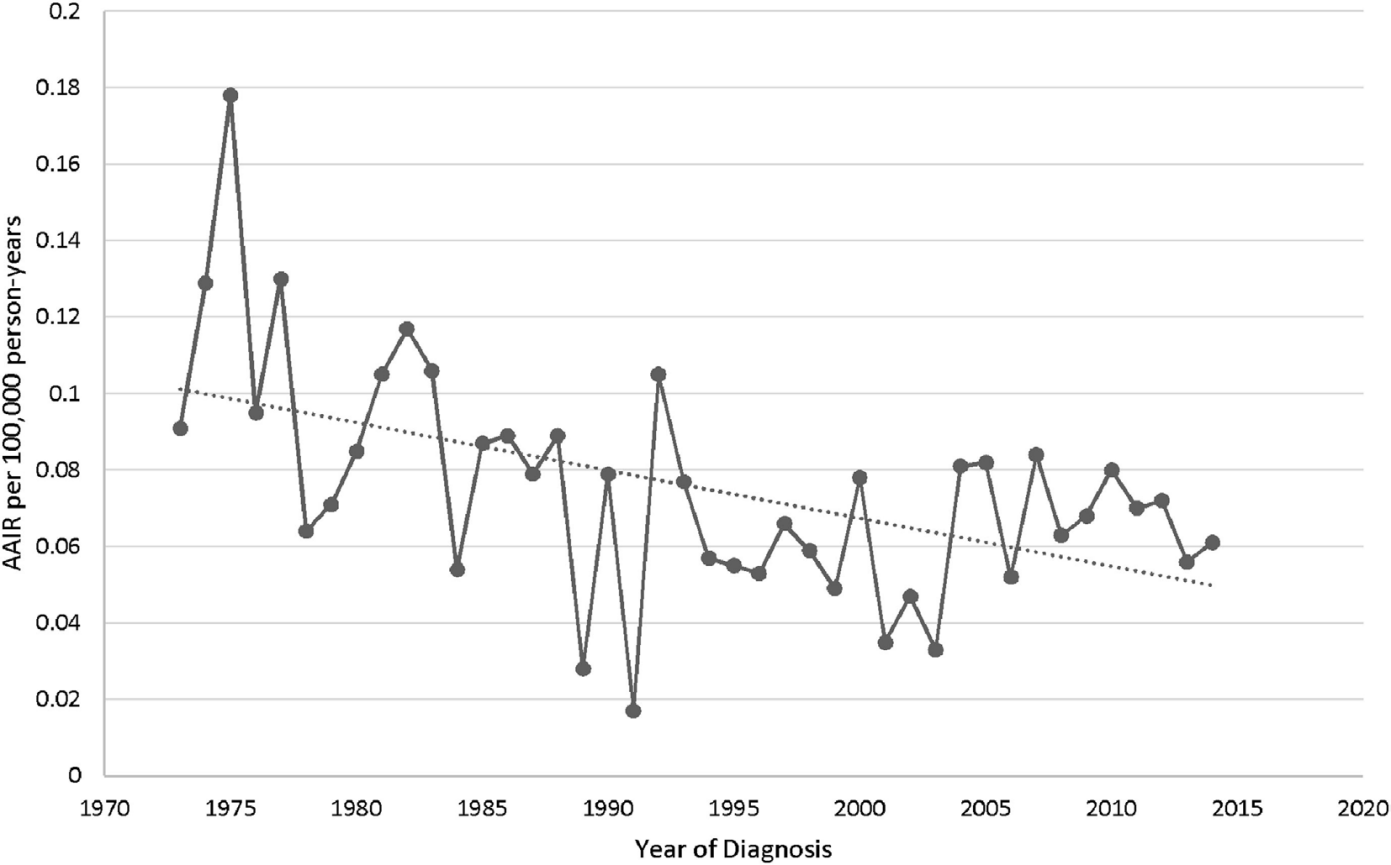
Trend in age-adjusted incidence rates of primary salivary gland tumors of lung in the United States.

Of 1,032 patients, 486 (46) were PACC and 546 (52) were PMEC (Figure 2). The median age and ethnic distribution of patients with PACC and PMEC did not differ. There was no sex predilection for PACC, whereas a slight female preponderance was noted for PMEC. However, the difference in the gender distribution between the two histopathologies was not statistically significant.

### Tumor Characteristics and Treatment Practices

The majority of the tumors were diagnosed in the lungs, 682 (66%) *p* ≤ 0.001. Significant heterogeneity was observed in the histopathologic subtypes and site of diagnosis, with 55% of PACC localized to the central airways (trachea and main bronchus) in contrast to 15% of PMEC (Figure 2; Table S1 in Supplementary Material). In the central airways, three-fourths (267/350) of the tumors were of PACC histopathology. In the lungs, two-thirds (463/682) of the tumors were of PMEC histology. The data for stage at the time of diagnosis were available for 711 (69%) patients in the SEER database. More than 50% patients had early stage (localized and regional) disease. A significantly greater number of patients with PMEC histology were diagnosed at an early stage than patients with PACC (62 vs. 48%; *p* = 0.02). The histopathologic grading was available for approximately 50% patients; for 113 (17) of adenoid cystic carcinoma (ACC) and for 392 (72%) patients with PMEC. Most of the patients in both histopathology groups, 86/113 (76) in PACC and 286/392 (73) in PMEC groups were diagnosed with low-grade tumors. Tumor size was reported for 577 (56) patients; 236 (49) PACC and 341 (62) PMEC. The majority of the advanced stage tumors (64%) were high grade when compared to those presenting with local and regional stages (*p* =≤ 0.001) (Table S2 in Supplementary Material). The median tumor size of PACC was significantly larger than PMEC (30 vs. 25 mm; *p* = 0.007).

Treatment data included information on surgery, radiation treatment, and chemotherapy. 467 (45%) patients underwent only surgical treatment while 218 (21%) also received adjuvant radiation (Figure 2; Table S3 in Supplementary Material). The most common surgical procedure was lobectomy in 64% of patients with PMEC and 36% in patients with PACC. Extensive surgery including complete resection and radical surgery was more often performed on patients with PACC (22%) when compared to patients with PMEC (10%). The rate of administration of adjuvant radiation therapy was significantly higher among patients with PACC when compared to patients with PMEC (*p* ≤ 0.001). Radiation as the only modality of treatment was employed in 172 (17%) patients without any difference between the two tumor types. The data on chemotherapy were available for 136 (13%) patients. 87% were coded as “no/unknown.” Out of 136 patients, only 43 received solely chemotherapy. There was no difference in the administration of chemotherapy to patients with either tumor type. A *post hoc* analysis was conducted to check for the stage shift across the study periods, as the year at the time of diagnosis was significantly associated with the outcome on multivariate analysis (see below). There was no stage shift across the study periods (*p* = 0.84) (Table S4 in Supplementary Material).

### Survival

**Figure 3:**
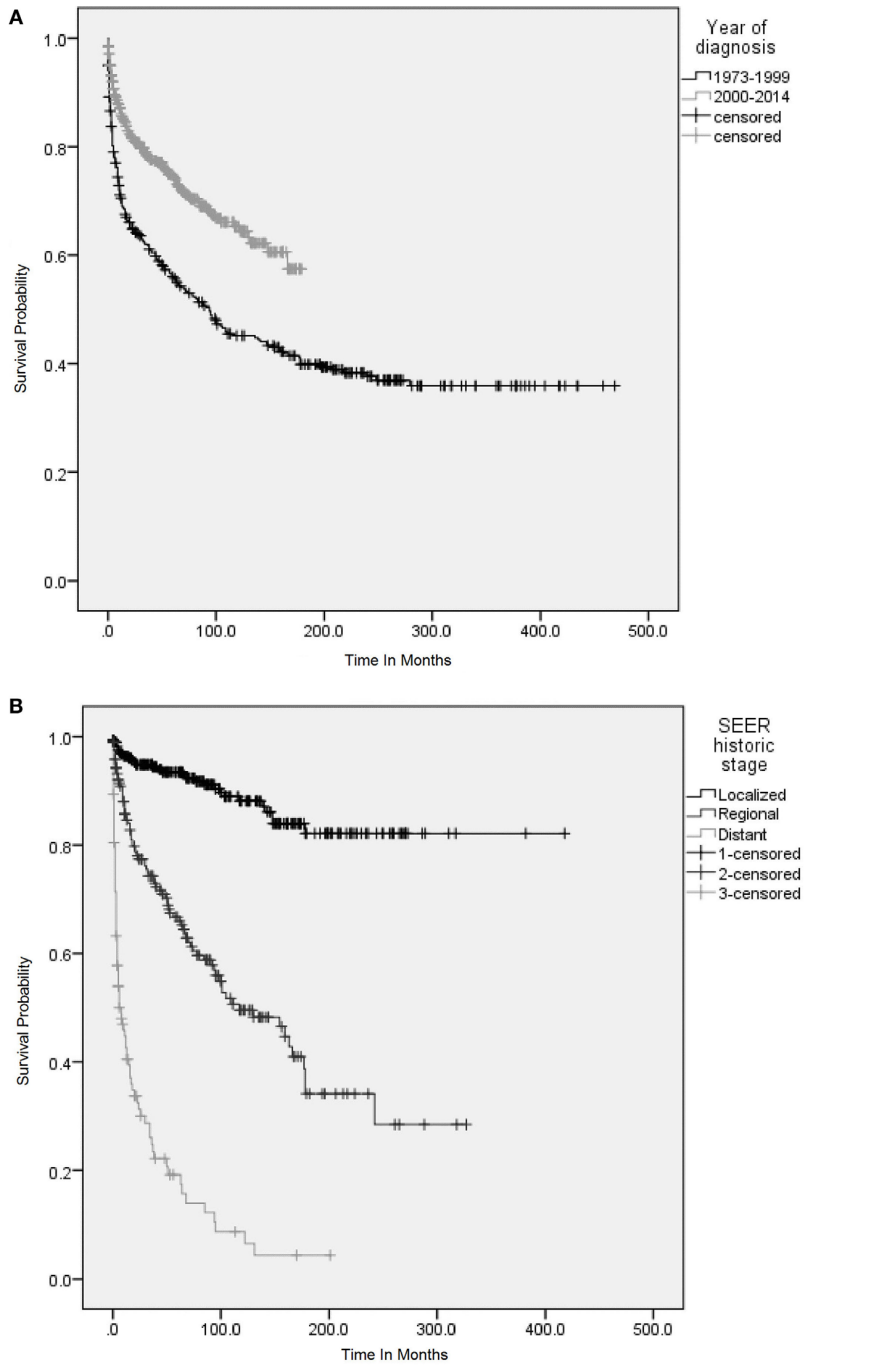

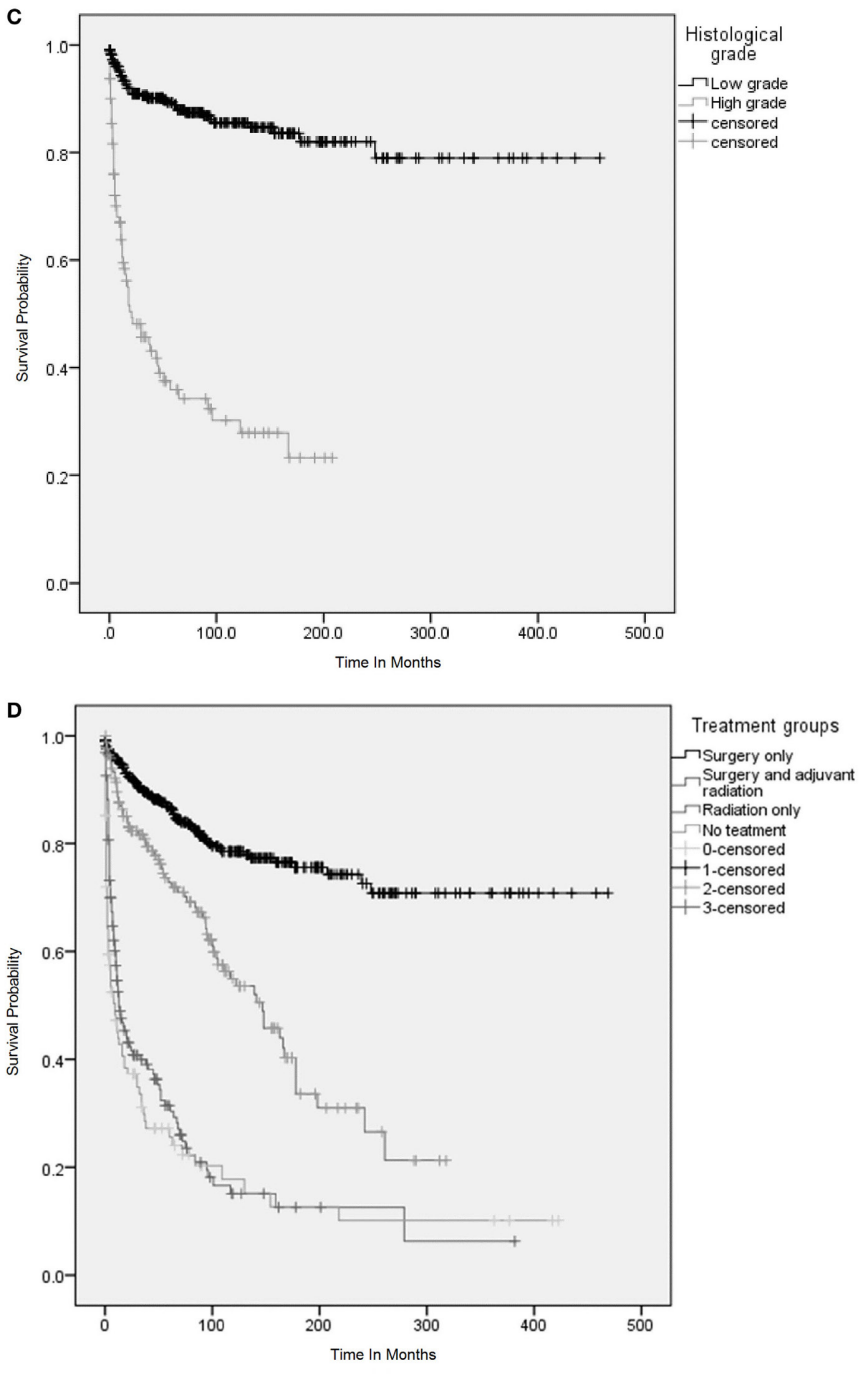
Overall survival (OS) was analyzed using Kaplan–Meier (KM) method and censored patients are indicated by vertical lines on the KM curves. **(A)** KM survival curves among patients with primary adenoid cystic carcinoma (ACC) and mucoepidermoid carcinoma (MEC) of lung according to the period of diagnosis. **(B)** KM survival curves according to stage (Surveillance Epidemiology and End Results staging) at diagnosis of primary AEC and MEC of lung. **(C)** KM survival curves according to the histological grades at diagnosis of primary ACC and MEC and **(D)** KM survival curves according to the treatment received. **(A)** OS was significantly higher among patients who were diagnosed during 2000–2014. Log rank *p* < 0.001. **(B)** OS among patients with localized disease was significantly higher as compared to patients with regional and distant metastasis. Log rank *p* < 0.001. **(C)** OS among patients with histologically low-grade disease was significantly higher as compared to patients with high-grade disease. Log rank *p* < 0.001. **(D)** OS among patients who underwent surgery only and adjuvant radiation was significantly higher as compared to patients who received only radiation or received no treatment. Log rank *p* < 0.001.

**Figure 4:**
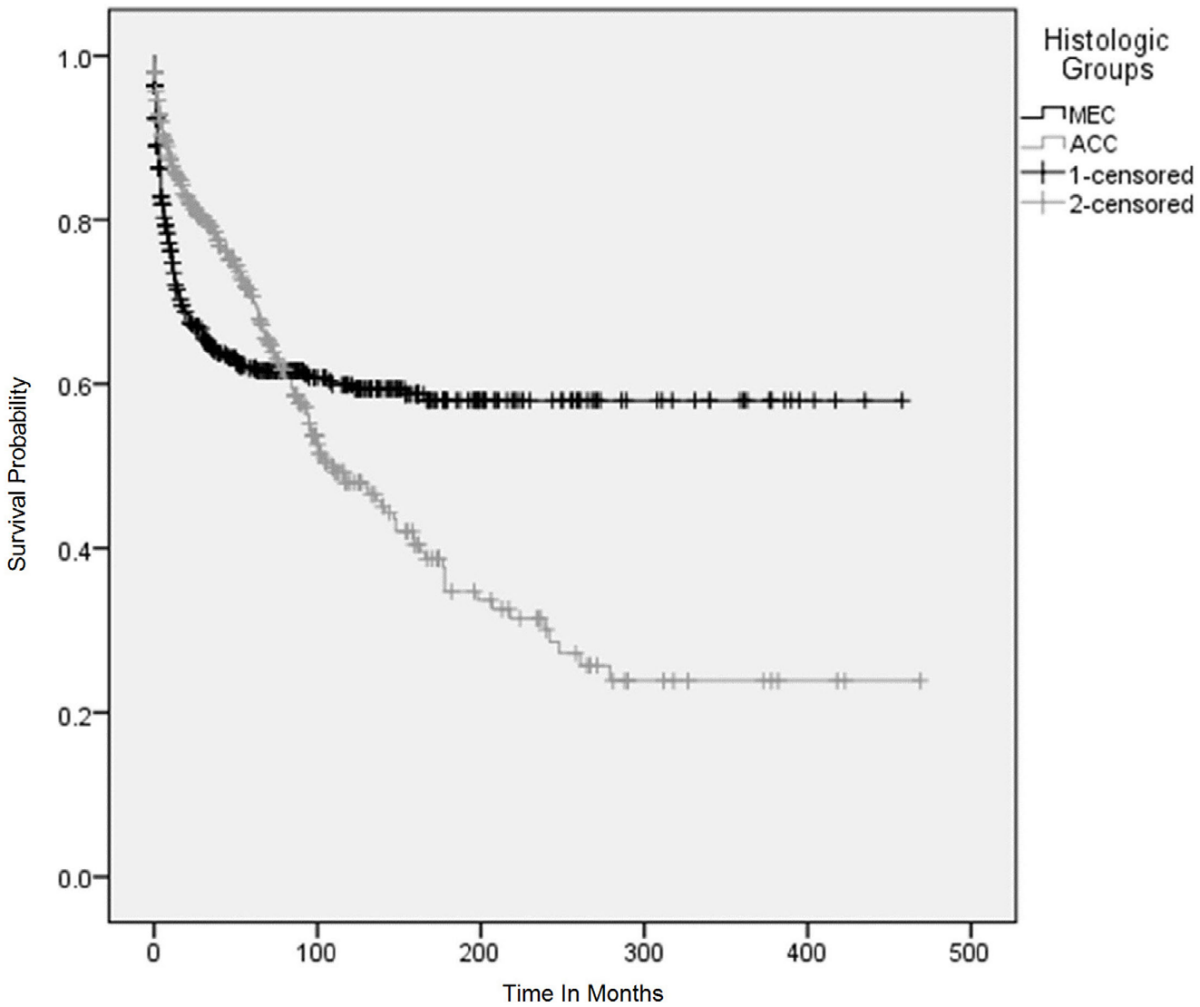
Overall survival using Kaplan–Meier (KM) method according to histological subtypes. The KM survival curves for patients with adenoid cystic carcinoma and mucoepidermoid carcinoma converged after 5 years and crossed each other between 7 and 8 years after the initial diagnosis and continued to diverge for 25 years and became parallel afterwards.

Survival analysis was conducted for 866 patients whose sequence of tumor was coded as one primary or first of 2 or more primaries. In the entire cohort of PSGT, the 1- and 5-year cause-specific survival was 76.6 and 62.8%, respectively. The 1- and 5-year cause-specific survival in various subgroups are shown in Table 2. K–M survival curves in selected subgroups are shown in Figures 3 and 4.

**Table 2:** 1- and 5-year cause-specific survival in selected subgroups of patients with primary adenoid cystic carcinoma (ACC) and mucoepidermoid carcinoma (MEC) of lung.

Subgroups	Cause-specific survival	Lower CI^a^	Upper CI	Cause-specific survival	Lower CI	Upper CI
						
	1-year (%)			5-year (%)		
Overall	76.6	72.5	80.2	62.8	58	67.2
**Gender**						
Female	81	75.1	85.7	67.4	60.5	73.4
Male	72.8	66.8	77.9	58.6	51.9	64.7
**Histological subtype**						
ACC	83.4	77.6	87.8	68.2	61.2	74.3
MEC	71	65	76.3	58.1	51.5	64.1
**Tumor grade**						
Low grade	92.5	86.9	95.8	88.1	81.5	92.4
High grade	59.8	45.2	71.7	34.8	21.4	48.6
**Stage**						
Localized	97.4	92.1	99.2	95.4	89.4	98.1
Regional	85.1	75.8	91.1	68.1	57	77
Distant	46.1	31.9	59.2	19.3	8.7	33.1
**Period of diagnosis**						
1973–1999	70.2	64.7	75.1	54.9	49	60.4
2000–2014	88.8	82.7	92.8	79	71.4	84.8
**Tumor site**						
Trachea	92.2	85	96	79.1	69.4	86
Lungs	71.3	65.8	76	56.1	50.1	61.6
Main bronchus and upper lobe	69.3	61.6	75.7	57.5	49.4	64.7
Middle and lower lobes	83.2	75.6	88.6	70.8	61.8	78.1
**Treatment**						
Surgery	92.8	87.4	95.9	86.2	79.3	90.9
Adjuvant radiation	85.1	73.2	91.9	72.7	58.3	82.8
Only radiation	60.1	50.7	68.2	40.5	31.6	49.1

*^a^Confidence interval: Log [−[log]] transformation. The level is 95%*.

#### Factors Associated With Survival

**Figure 5:**
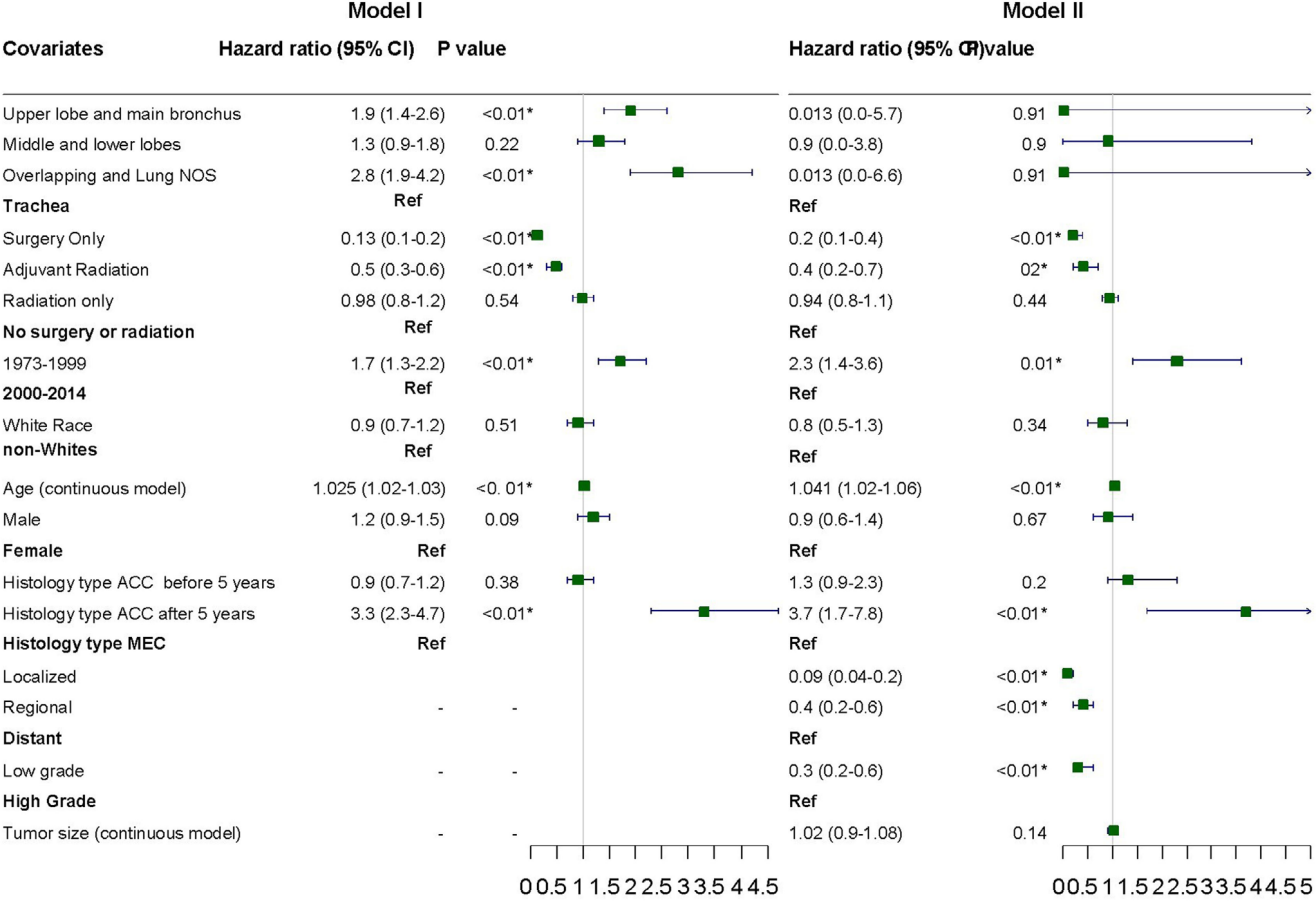
Hazard ratios with 95% confidence intervals for predictors of adverse outcome on cox proportional hazard analysis.

Variables were adjusted in stepwise models as described above in the section on statistical analysis due to missing information on a considerable number of patients. Surgery with or without adjuvant radiation, year of diagnosis, and age were consistently significant for adverse outcome across the various models (Figure 5). Stage and tumor grades were also significant predictors of outcome after adjusting for other factors in the final model. The HR for PACC histology compared to PMEC did not differ significantly in the first 5 years. However, the difference in HR became apparent after 5 years at which point the HR for adverse outcome was 3.3 times higher in patients with PACC compared to the PMEC in model I. This difference persisted after adjusting for other variables in model II (Figure 5).

## Discussion

This large population based study demonstrates that PACC and PMEC are rare tumors with combined United States incidence of 7 cases per 10 million person-years. Overall, the two major forms constituted ~0.09% of all lung tumors during the period 1973–2014. In this analysis, PMEC was slightly more common than PACC. Though differences in the demographic characteristics of patients with PMEC and PACC were not observed, differences in the tumor histopathologic characteristics, treatment practices, and long-term survival were notably present. As expected, age, stage at the time of diagnosis, histopathologic grade, period of diagnosis, and surgical treatment were the predictors for outcome on multivariate analysis. Tumor histopathology demonstrated a strong interaction with time and had an impact on survival independently only after 5 years.

Primary salivary gland-type tumor constituted <1% of all lung tumors in small retrospective studies (2, 3). The present study reinforces these observations with the two major histopathological subtypes constituting 0.087% of all lung tumors. The combined incidence of two most common PSGTs in the United States is 7 cases per 10 million population. Moreover, the incidence is decreasing with annual percent change of 1.6%. The underlying reason for the decrease in the incidence of PSGT is not known. Improvements in imaging and histopathologic analysis may have established that many salivary tumors which were thought to be primary actually represented metastases from the salivary glands. Hsu et al. reported that most of the solitary PACC present in the periphery of the lungs actually metastasized from the salivary glands. They suggested that extra thoracic primary sites should be searched for when solitary pulmonary lesions are identified (18).

Pulmonary adenoid cystic carcinoma and PMEC are the two most common forms of PSGTs with PMEC considered to be more common than PACC (2, 11, 18, 19). This study supports the slightly higher incidence of PMEC compared to PACC. No differences were identified in the demographic profiles of patients with PACC and PMEC. The age range of patients with PACC and PMEC has been reported to be 21–80 and 3–78 years, respectively, with PMEC affecting patients in younger age group as compared to PACC (10, 17, 20–28). In this study, the median age of patients in both the groups was 57 years. Though one-quarter of patients with PMEC were younger than 37 years, the difference in the age distribution was not statistically different in the two groups. The current literature suggests female predilection or no gender predisposition for patients with PACC, and male predominance or no gender predisposition for PMEC (11, 18, 24). In this analysis, there was no gender predilection for PACC while slight female preponderance was seen in the patients with PMEC, similar to the findings of Yamamato et al. However, the difference in the gender distribution in the two groups was not statistically significant (29).

Pulmonary adenoid cystic carcinoma was mostly localized to the trachea and main bronchus while PMEC was more commonly diagnosed in the lung. Tumor size data was available for approximately one-half of the patients and the tumors of PACC histology were significantly larger than PMEC, as has been observed in prior studies (2, 14, 18). The PSGT are often low-grade tumors and metastases are uncommon at the time of presentation (11). In our study, approximately 74% of the tumors were well or moderately differentiated on diagnosis. There was no difference in the stage and grade at the time of diagnosis between the two types. On further analysis, the majority of advanced stage tumors were poorly differentiated, a finding which has also been suggested in other studies (16, 18).

The survival of patients with PSGT has been reported to be significantly better than that for patients with non-small cell lung cancer (NSCLC) (19). However, survival of patients with PACC was reported to be similar to that of patients with NSCLC (26).

The 5-year survival in PSGT has been reported to be 65% in the literature (11). In our study, 1- and 5-year survival was 76 and 63%, respectively, similar to other studies. Several factors independently affected survival as demonstrated by cox multivariate analysis including age at the time of diagnosis, stage, histological grade, time period when the diagnosis was made, and surgical treatment. Poor outcomes with advanced age and stage has been reported previously in small studies (11, 17, 19, 21, 25). The influence of histopathologic grade and surgery on outcome has also been reported previously (17, 21). Yousem et al. reported survival of 95% in 41 patients with low-grade PMEC vs. 75% in patients who had high-grade tumors, and they noted parenchymal invasion in approximately 50% of the patients with high-grade tumors (30). Tumor grade has also been suggested as a guide for the treatment of patients with PMEC. Conlan et al. recommended surgery for grade I tumors, while adjuvant therapy was suggested for patients with grade II and III PMEC (19). Alternatively, the impact of tumor grade on prognosis has also been reported for patients with PACC. The grading system of PSGT is evolving. The grading of PMEC is complicated and based upon cytology and cellular composition, whereas the grading of PACC is based on predominant growth pattern (9, 31). The PACC with tubular pattern are graded as I while cribriform and solid are graded as II and III, respectively (31). This grading system was not developed in the past and SEER used grading system based on cellular differentiation. Our study did not reveal a difference in the survival of well and moderately differentiated tumors, thus they were included together as low grade. A correlation between tumor stage and grade was observed with distant metastatic tumor being predominantly high grade, which has also been reported previously (11).

Survival was also affected by surgical treatment with lower HRs for deaths in the patients who underwent surgery. Differences in treatment practices in the United States were noted with significantly higher number of patients with PACC undergoing extensive surgery compared to those with PMEC. Difficulties in the surgical management of patients with PACC include large size, and adverse location, not readily amenable to surgery, i.e., distal trachea and carina (18, 32–34). PACC is often more infiltrative, invading surrounding structures, necessitating extensive surgery (35). Intraoperative frozen section histopathologic analysis has demonstrated that surgical margins are frequently positive, indicating incomplete resection, attributable to the erratic pattern of extension (36). However, several studies have suggested that survival does not differ in patients with complete resection compared to the patients with residual tumor (37, 38). In one study, the 5-year survival was 82 and 77% in the patients who had complete and incomplete resection (37). To reiterate, surgery is also the treatment modality of choice for patients with PMEC (39). Patients who underwent surgery had better survival compared to the patients without surgery (11).

The rate of administration of adjuvant radiation was higher in patients with PACC compared to patients with MEC. The administration of adjuvant radiotherapy prolonged survival compared to without surgery which is in concordance to the previous studies (40). Adjuvant radiotherapy has been advocated for the patients with PACC who have residual tumor or positive margins (10, 41). In patients with PMEC, the use of adjuvant therapy has been suggested for those with grade II and III tumors (19). Although the HR for patients who received adjuvant radiation was higher than the patients who were treated with surgery alone, after adjusting for other variables the difference in the HR was not significant.

The impact of histopathology on survival was complex and demonstrated a strong interaction with time on CPH analysis in this study. After adjusting for demographic factors and site of tumor (data points which were available for most of the patients), the HR was not different in the patients with PACC histology as compared to PMEC in the first 5 years. However, after 5 years, HR changed in favor of patients with PMEC compared to those with PACC, who had a 3.3 times higher risk of dying after 5-year survival This effect persisted in model II after adjustments made for stage, tumor grade, and size. The poor long-term outcome for PACC has been attributed to the presence of micro invasions leading to higher rates of positive tumor margins, which in turn increase the chances of recurrences and metastases (42). Despite this, no differences in overall survival were reported by ElNayal et al. (14). However, effect of histopathology on survival was not assessed by multivariate analysis in that study. Other study demonstrated that patients with PMEC, especially low-grade tumors, had a better outcome (11).

Tumor diagnosis in the period after year 2000 was an independent predictor of better outcome when compared to the diagnosis in the earlier period. On secondary analysis, there was no “stage shift” in the more recent study period. In fact, this is more likely due to improvement in the techniques of thoracic surgery for tumor resection and anastomosis utilizing artificial prostheses and graft transplantation, leading to better airway reconstruction (26, 42). The improvement in outcome over time has also been reported by Hsu et al. (18).

The role of chemotherapy is not clearly defined in the patients with PSGT. Chemotherapy is employed for palliation in these patients (18, 43). The chemotherapy was not included in multivariate analysis because this information was available only for a small percentage of patients (~15%). Furthermore, most of the patients who received chemotherapy also underwent surgery and/or radiation therapy thus it was not possible to dissect the effect of other two modalities from chemotherapy in these patients. Moreover, the information was available as “yes” and “no/unknown” making it impossible to assess the patient adherence.

The strength of the current study includes large sample size and the fact that it is derived from a publicly available population based registry, which is reflective of the survival of patients in the “real world.” Data on survival duration and mortality were available for most of the patients. Nevertheless, findings in this data set analysis should be interpreted in the light of following shortcomings. The data on tumor stage and histological grade were not available for a considerable number of patients restricting the power of this study. Despite this, more than 50% patients had data on both variables; thus, estimation of their impact on survival is reliable. The grading system used in SEER is based on cellular differentiation; however, the grading system of PSGT is evolving and is quite complex particularly for PMEC. The data on tumor stage were available as SEER stage (local, regional, and distant) instead of the more frequently reported tumor, node, metastasis staging. The data on chemotherapy were only available for a small number of patients (~15%). Additionally, the information on chemotherapy was available as “yes” and “no/unknown.” The other factors related to chemotherapy which could affect outcome, e.g., patient adherence, could not be calculated. We assessed cancer-specific survival based on cause of deaths derived from death certificates. Though the SEER database accurately records the cause of death, misclassification is possible in some cases. However, due to the large sample size, the impact of this on overall analysis would be small.

Despite these shortcomings, the data from current analysis would contribute to our understanding on the epidemiology and factors affecting survival in these rare tumors.

## Conclusion

This study demonstrates that PSGT of lungs are rare tumors whose incidence is decreasing in the United States in the recent times. The incidence rate of PMEC is slightly higher than PACC. There is no significant difference in the demographic characteristics of patients with PACC and PMEC. However, significant differences were noted in the tumor characteristics. Several factors such as age at the time of diagnosis, stage, histological grade, surgery, and time period of diagnosis affected survival. Tumor histopathology affected survival only after 5 years.

## Ethics Statement

The Maimonides Medical Center Institutional Review Board (IRB) has deemed that studies which utilize de-identified, publicly available data (current study) to be exempt from IRB review.

## Author Contributions

VK: data collection, analysis, and manuscript writing. AC and PS: result interpretation, manuscript writing, and proofreading. AG: manuscript writing and proof reading. MG: literature search, proof reading, and manuscript writing. MT: manuscript writing, proof reading, and analysis. SK: mentoring of project, manuscript writing, and proof reading.

## Conflict of Interest Statement

The authors declare that the research was conducted in the absence of any commercial or financial relationships that could be construed as a potential conflict of interest.

## Abbreviations

PSGT, primary salivary gland-type tumor; PACC, pulmonary adenoid cystic carcinoma; PMEC, pulmonary mucoepidermoid carcinoma; SEER, Surveillance Epidemiology and End Results; HR, hazard ratio; NSCLC, non-small cell lung cancer; WHO, World Health Organization.
