# Supplementary material for: A Comparative Study of Primary Adenoid Cystic and Mucoepidermoid Carcinoma of Lung
Source: Front Oncol. 2018 May 15;8:153. doi: 10.3389/fonc.2018.00153 (PMC5962707; doi:10.3389/fonc.2018.00153)
Supplement: Supplementary file 1 [file data_sheet_1.DOCX]

**Table E1. Tumor location in detail for patients with ACC and MEC**

|  | **ACC** | **MEC** |
| --- | --- | --- |
| **Tumor characteristics** |  |  |
| *Trachea* | **192(40)** | **25(5)** |
| ***Main bronchus***  *Right*  *Left*  *Unknown* | **72(15)**  27(6)  38(8)  7(1) | **58(11)**  25(5)  32(6)  1(<1) |
| **Lungs** | **222 (45)** | **463(85)** |
| ***Upper lobe***  *Right*  *Left*  *others* | 82(17)  46(9)  36(8)  0(0) | 192(35)  87(16)  103(19)  2(<1) |
| ***Middle lobe***  ***Lower lobe***  *Right*  *Left*  *others* | 16(3)  87(18)  47(10)  39(8)  1(<1) | 47(9)  152(28)  71(13)  80(15)  19(<1) |
| ***Overlapping and lung NOS***  *Right*  *Left*  *others* | 37(7)  15(3)  12(2)  10(2) | 72(13)  44(8)  17(3)  11(2) |

**Table E2. Distribution of tumor grades according to stage of tumor on diagnosis.**

|  | **Localized**  **240 (100)** | **Regional**  **113 (100)** | **Distant**  **50 (100)** | ***‘P’* value** |
| --- | --- | --- | --- | --- |
| Well Differentiated | 85 (35) | 28(25) | 9(18) | <0.001 |
| Moderately Differentiated | 126(53) | 49(43) | 9(18) |  |
| Poorly Differentiated/anaplastic | 29(12) | 36(32) | 32(64) |  |

**Table E3. Types of surgery**

| **Treatment** | **ACC**  **(319)** | **MEC**  **(366)** |
| --- | --- | --- |
| Local ablation and partial resection | 69(22) | 55(15) |
| Lobectomy/Bilobectomy | 115(36) | 235(64) |
| Complete resection | 33(10) | 16 (4) |
| Radical surgery | 39(12) | 23(6) |
| Surgery NOS | 63(20) | 37(10) |

**Table E4. Stage-wise distribution of patients with PSLT across the study period.**

| **Stage** | **1973-1999**  **N= 392** | **2000-2014**  **N=640** | ***‘P’ value*** |
| --- | --- | --- | --- |
| Localized | 89 (23) | 255 (40) | 0.84# |
| Regional | 64 (16) | 165 (26) |  |
| Distant | 38 (10) | 100 (15) |  |
| Unknown | 201(51) | 120 (19) |  |

**#p value after excluding patients of ‘unknown stage’**
